# Visual Motion Prediction and Verbal False Memory Performance in Autistic Children

**DOI:** 10.1002/aur.1915

**Published:** 2017-12-21

**Authors:** Furtuna G. Tewolde, Dorothy V. M. Bishop, Catherine Manning

**Affiliations:** ^1^ Department of Experimental Psychology University of Oxford Oxford UK

**Keywords:** autism, motion perception, visual perception, memory, child development, neurodevelopmental disorders

## Abstract

Recent theoretical accounts propose that atypical predictive processing can explain the diverse cognitive and behavioral features associated with autism, and that difficulties in making predictions may be related to reduced contextual processing. In this pre‐registered study, 30 autistic children aged 6–14 years and 30 typically developing children matched in age and non‐verbal IQ completed visual extrapolation and false memory tasks to assess predictive abilities and contextual processing, respectively. In the visual extrapolation tasks, children were asked to predict when an occluded car would reach the end of a road and when an occluded set of lights would fill up a grid. Autistic children made predictions that were just as precise as those made by typically developing children, across a range of occlusion durations. In the false memory task, autistic and typically developing children did not differ significantly in their discrimination between items presented in a list and semantically related, non‐presented items, although the data were insensitive, suggesting the need for larger samples. Our findings help to refine theoretical accounts by challenging the notion that autism is caused by pervasively disordered prediction abilities. Further studies will be required to assess the relationship between predictive processing and context use in autism, and to establish the conditions under which predictive processing may be impaired. ***Autism Res*** *2018, 11: 509–518*. © 2017 The Authors Autism Research published by International Society for Autism Research and Wiley Periodicals, Inc.

**Lay Summary:**

It has been suggested that autistic individuals have difficulties making predictions and perceiving the overall gist of things. Yet, here we found that autistic children made similar predictions about hidden objects as non‐autistic children. In a memory task, autistic children were slightly less confused about whether they had heard a word before, when words were closely related in meaning. We conclude that autistic children do not show difficulties with this type of prediction.

## Introduction

Many theories have linked autism to cognitive impairments, including weak central coherence [Frith, 1989; Frith & Happé, 1994], impaired executive functioning [Pennington & Ozonoff, 1996] and reduced theory of mind [Baron‐Cohen, Leslie, & Frith, 1985]. Recently the focus has turned to atypical predictive processing as a potential cause of social and non‐social symptoms, including altered sensory processing, insistence on sameness, repetitive behaviors and difficulty with social interactions [Friston, Lawson, & Frith, 2013; Gomot & Wicker, 2012; Lawson, Rees, & Friston, 2014; Palmer, Lawson, & Hohwy, 2017; Pellicano & Burr, 2012; Sinha et al., 2014; van de Cruys et al., 2014]. Pellicano and Burr [2012] proposed that autistic individuals use prior information less than neurotypical individuals, potentially hindering predictions about future events. Related accounts situated in a predictive coding framework focus on the difference between predictions and what actually happens, and how these ‘prediction error’ signals are treated. Van de Cruys et al. [2014] suggested that the ability to generate predictions is preserved in autism, but that prediction errors are given uniformly, inflexibly high weighting. Lawson and colleagues also suggested that prediction errors may be weighted more highly in autism, either as a result of over‐precise estimates of sensory precision or under‐precise estimates of prior precision [Lawson et al., 2014; Palmer et al., 2017]. The predictive coding framework is hierarchical, with prediction errors passed up the hierarchy to inform higher‐level expectations, while the nervous system works to minimize prediction error across the whole system. Therefore, it is possible that the weighting of prediction errors may vary at different levels of representation [Palmer et al., 2017]. Finally, Sinha et al. [2014] suggested that autism is characterized by generally disordered prediction, stemming from impairments in estimating temporally unfolding Markov systems, with autistic individuals having difficulties detecting the conditional probability of event B occurring, given the occurrence of event A.

While varying in instantiation, these accounts all suggest that predictive processing is atypical in autism. Reports of reduced habituation or adaptation [Kleinhans et al., 2009; Pellicano, Jeffery, Burr, & Rhodes, 2007; Turi et al., 2015; Lawson, Aylward, White, & Rees, 2015] and dampened responses to unexpected stimuli [Lawson, Mathys, & Rees, 2017; Dunn et al., 2008] are potentially consistent with impaired predictions, in line with Bayesian and predictive coding accounts. However, these studies do not directly assess Sinha et al.'s claim of difficulties making predictions in autism. Studies using more direct tests have provided inconsistent results. Sheppard, van Loon, Underwood, and Ropar [2016] reported that young autistic adults were less accurate at judging whether they or another car would reach a junction first in a simulated driving scenario, but only for straight and not curved roads. Similarly, Schuwerk, Sodian, and Paulus [2016] showed that autistic children and adults were less likely to predict the repeated movement of an occluded agent, as measured using proactive eye movements. In contrast, Ego et al. [2016] showed typical anticipatory eye movements in autistic populations suggesting preserved prediction abilities.

Given the plurality of theoretical accounts and mixed empirical findings, the nature of predictive processing in autism is unclear. It is unlikely that all prediction abilities are impaired. Various authors have noted that autistic individuals tend to perform well when dealing with predictive relationships that are lawful or deterministic [Baron‐Cohen, 2002, 2006; Mottron et al., 2013; Gomot & Wicker, 2012; Sinha et al., 2014 Van de Cruys et al., 2014] and when making predictions about self‐generated actions. Indeed, in his early writings, Kanner [1943] noted that an autistic child may be perturbed by loud external noises, yet be unperturbed by loud self‐generated noises. Likewise, autistic individuals show an attenuated tickling response when tickling themselves, like those without autism [Blakemore et al., 2006], and appear to have preserved predictive motor control, although the research evidence is mixed [Gowen & Hamilton, 2013]. This distinction between external and self‐generated events could explain why autistic individuals often engage in repetitive behaviors, potentially allowing them to minimize prediction error [Lawson et al., 2014; van de Cruys et al., 2014]. Additionally, predictive impairments may be particularly pronounced in uncertain situations, such as social scenarios [Gomot & Wicker, 2012; Lawson et al., 2014].

In this study, we focused on predictions by Sinha et al. [2014] that prediction impairments should be manifest in a range of domains, including the ability to interact with dynamic objects, due to difficulties keeping track of and anticipating object motion. Sinha et al. proposed that impairments should be most evident for predictions where the relationship between events is probabilistically weak (i.e., where event A does not consistently precede event B) or where the events are separated by a long time. Here we used two dynamic extrapolation tasks to investigate how well autistic children can predict the end‐point of occluded dynamic events. Performance in these tasks is thought to involve a domain‐general rate control system for updating the mental representations of occluded objects [Makin & Bertamini, 2014]. In one task, children were asked to predict the end‐point of an occluded target moving horizontally, and in the other, children were asked to predict the end‐point of accumulating elements. In terms of Sinha et al.'s operationalization, the relationship between unfolding events in our tasks was strong, as the dynamic objects moved at a consistent rate. However, we manipulated difficulty by varying the occlusion duration of events. We therefore expected difficulties to be more pronounced at the longest occlusion duration.

It is not always clear from prediction accounts what the scope of 'prior information' is. Tasks using occlusion investigate prediction in a specific task over a short time scale. But in everyday life, we continually make predictions based on knowledge acquired over a lifetime. If a child fails to extract key information to generalize about how objects and people behave from prior experience, then they may fail to predict accurately. This could relate to an inability to take context into account [e.g., Gomot & Wicker, 2012; Lawson et al., 2014] and/or generalize to new situations [van de Cruys et al., 2014], which are key claims of weak central coherence [Frith & Happé, 1994] and reduced generalization theory [Plaisted, 2001]. Failure to generalize can sometimes lead to superior task performance in situations where it is important to remember specific detail rather than more global information. An example is the false memory illusion task [Roediger & McDermott, 1995] in which participants falsely remember an item (e.g., word) that was not presented in a list, due to its close semantic relationship with presented list items. Autistic adults have been shown to be less susceptible to false memory illusions [Beversdorf et al., 2000; Hillier, Campbell, Keillor, Phillips, & Beversdorf, 2007; Kamio & Toichi, 2007; Parra et al., 2016; but see also Bowler, Gardiner, Grice, & Saavalainen, 2000], and this has been attributed to a reduced reliance on ‘gist’ or contextual processing [Miller, Odegard, & Allen, 2014]. We therefore reasoned that autistic children would show superior false memory performance. If compromised prediction abilities in autism are linked to reduced contextual processing, then performance in the false memory and extrapolation tasks should be related.

To summarize, we presented dynamic extrapolation and false memory tasks to autistic and typically developing children and hypothesized that, (a) autistic children would show less precise (more variable) predictions in both dynamic extrapolation tasks, with a widening gap in performance as occlusion durations increase, (b) autistic children would be less susceptible to false memories, with a greater sensitivity for discriminating true items from false items and recognizing fewer critical lures compared to typically developing children, and (c) reduced prediction precision would be related to increased sensitivity in the false memory task.

## Methods

### Ethical Approval and Preregistration

The study was approved by the Central University Research Ethics Committee in line with the Declaration of Helsinki. Informed written consent was obtained from parents and written assent was given by children. The hypotheses and procedure were pre‐registered on the Open Science Framework prior to data collection: https://osf.io/pv4w6/register/565fb3678c5e4a66b5582http://f67. Data and analysis scripts, as well as minor changes regarding typographical errors and clarifications to the preregistration document, can be found here: https://osf.io/kwdjs/.

### Participants

We conducted an a priori power analysis using G*Power software [Faul, Erdfelder, Lang, & Buchner, 2007] with an effect size of *d* = 0.65 based on Hillier et al.'s [2007] study of false memory performance in autistic and control populations, in the absence of prior data for the visual extrapolation tasks. Thirty participants in each group give 80% power with an alpha‐level of 0.05 in a one‐sided test. Accordingly, we recruited 30 typically developing (16 female) and 30 autistic children (25 female) aged 6–14 years with normal or above‐normal intellectual ability (IQ > 70) and normal‐ or corrected‐to‐normal visual acuity (assessed using a Snellen chart).

The typically developing children had no history of neurodevelopmental disorders and scored below the autism cut‐off of 15 on the Social Communication Questionnaire [SCQ; Rutter, Bailey, and Lord, 2003]. The autistic children had an independent diagnosis of an autism spectrum condition, and met criteria for an autism spectrum condition in the Autism Diagnostic Observation Schedule‐2 [ADOS‐2; Lord et al., 2012] and/or the SCQ (n.b. three and five children did not meet criteria on the SCQ and ADOS‐2, respectively). Intellectual ability was quantified using the Wechsler Abbreviated Scales of Intelligence, 2nd edition [WASI‐2; Wechsler, 2011]. The groups of children did not differ significantly in age, *t*(58) = 1.21, *P* = 0.23, or performance IQ, *t*(58) = .48, *P* = 0.63. However, the autistic children had lower verbal IQ scores than the typically developing children, *t*(58) = 2.33, *P* = 0.02 (see Table 1 for descriptive statistics). We did not aim to achieve gender‐matching in our sample, but considered this factor in our analyses.

**Table 1:** Participant Demographics

	Typically developing			Autistic		
Measure	*M*	SD	Range	*M*	SD	Range
Age	10.48	2.18	6.08–14.03	11.16	2.23	7.43–14.95
Verbal IQ	112.17	13.40	83–138	104.23	12.96	76–131
Performance IQ	107.93	16.03	77–147	105.93	16.18	74–138
Full‐scale IQ	111.40	14.74	78–143	105.67	15.32	74–130
SCQ	3.67	3.93	0–14	22.27	6.09	6–32
ADOS‐2 Severity Score	N/A			6.80	2.44	1–10

*Note*. SCQ = Social Communication Questionnaire [Rutter et al., 2003]. ADOS‐2 = Autism Diagnostic Observation Schedule‐2 [Lord et al., 2012]. Severity scores are reported for the ADOS‐2 to allow comparability across modules [Lord et al., 2012; Hus & Lord, 2014].

### Apparatus, Stimuli and Materials

**Figure 1:**
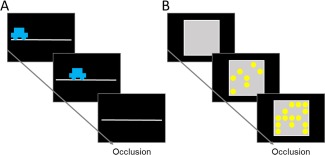
Schematic representation of stimuli in visual extrapolation tasks. (**A**) In the position extrapolation task, a car moved along a track until it became occluded (or invisible). Children were asked to press a button when the invisible car would have reached the end of the track. (**B**) In the accumulation extrapolation task, a grid filled with yellow lights sequentially in random grid positions until the point of occlusion, when no more lights turned on. Children were asked to press a button when the lights would have all turned on if they had not been broken. Note that the lights that had already appeared remained on the screen until the child made a response. This figure is not drawn to scale.

Visual extrapolation tasks were presented on a Dell Inspiron 13 7000 laptop (1920 × 1080 pixels; 60Hz) using PsychoPy [Peirce, 2007], with stimuli presented on a black background. In the position extrapolation task, the stimulus was a small blue car (1.20° × 0.75°) moving along a grey track (length 20°). In the accumulation extrapolation task, the stimulus was a grey square (5° × 5°) that filled with yellow Gaussian‐edged circles (‘lights’) in a grid layout, with space for 25 ‘lights’ in total (Fig. 1).

The false memory task was presented verbally using word lists modified from a previous study of false memory in children [Metzger et al., 2008]. There were six lists of eight words that were semantically related to each other and to a critical lure that was not presented. The recognition test consisted of seven items: two items previously listed (“true”), two items distantly related to the listed items but not listed (“distant false”), two items unrelated to the previous list and not listed (“unrelated false”) and the critical “lure” item which was not listed but strongly semantically related to the list content (see Supporting Information).

### Procedure

Children completed two visual extrapolation tasks, separated by the false memory task, in a session lasting approximately 30 min. The order of the visual extrapolation tasks (position or accumulation first) was counterbalanced among participants. The tasks were presented as circus‐themed games, and children collected stickers for completing ‘levels’. Children sat at a viewing distance of 30 cm from the screen. The WASI‐2, ADOS‐2 and Snellen test were completed in further sessions.

### Visual Extrapolation Tasks

The visual extrapolation tasks were adapted from Makin and Bertamini [2014] for use with children. In the position extrapolation task, children saw a car moving along a track until it became occluded and pressed a button when they thought the car would have arrived at the end of the track, had it not been occluded. Children were told that the cars were being made invisible by a jester, and that they should help the circus work out when the invisible cars arrived. The car's direction (leftwards/rightwards) was randomized across trials.

In the accumulation task, children viewed a grid that was filled gradually with lights, appearing sequentially in random locations, until the lights stopped accumulating (i.e., became occluded). Children were asked to determine when the grid would have filled up with lights, had the lights continued to accumulate. Children were told that some of the circus lights were broken and that they needed to press a button when the lights should have all turned on, so that the show could start. Note that the size of the grid was smaller than that used by Makin and Bertamini [2014] to avoid a floor effect in children's performance.

The movement speed (the rate of movement of the car along the track or the rate of accumulation of lights) and the point at which the occlusion started was varied across trials. These factors jointly determined the occlusion duration. Each task consisted of 60 trials. Twelve of these were ‘filler’ trials with randomly selected movement speeds and points of occlusion, which were not analyzed but were included to minimize overlearning the occlusion durations in the experimental trials. In the experimental trials, the occlusion duration was either 1000 ms, 2000 ms or 4000 ms, with 16 trials for each duration. These occlusion durations were achieved either by (a) fixing the start‐point of occlusion (at 60% of the track/total lights) and manipulating the movement speed (10%, 20% or 40% of the track/total lights per second) or, (b) fixing the movement speed (at 20% of the track/total lights per second) and manipulating the point of occlusion (20%, 60% or 80% of the track/total lights). An equal number of trials for each trial type were presented. The trials were divided into six ‘levels’. The reaction times (RT) of children's button presses were recorded.

Children were presented with animated explanations for each task in a familiarization phase. They watched the experimenter complete two demonstration trials before completing six practice trials themselves. The experimenter repeated the practice trials for one participant to ensure task understanding.

### False Memory Task

The experimenter read out each word list, which children were asked to remember, followed immediately by a verbal recognition test in which children confirmed or denied whether each test word was presented in the previous list. The list presentation was counterbalanced by cycling through six different orders of list arrangement. The position of the critical lure in the recognition test was randomized between the 5th, 6th, and 7th place. Children's responses were recorded manually.

### Data Processing

Trials from the visual extrapolation tasks with very short (≤ 300 ms) or long (≥ 8000 ms) RTs were excluded from analysis. On average, 99% (range 88–100%) of each participant's data was retained in the position task and 98% of each participant's data (range 77–100%) was retained in the accumulation task, and no participants were excluded from analysis. We computed the median RT, the median RT error (RT – correct RT) and the standard deviation of the RT error for each participant, for each occlusion duration. Outliers were defined as data points lying 3 or more standard deviations from the group mean for each occlusion duration and measure. Five outlying points were identified in the autism group, and 6 in the typical group, which were replaced with data points at 2.5 standard deviations from the group mean in the same direction as the original data point [Tabachnick & Fidell, 2007].

For the false memory task, our primary measure was *d*‐prime (*d*′) reflecting the discrimination of true listed items versus the non‐listed lure items, pooled across lists (note that this measure was normally distributed so we did not use the non‐parametric equivalent). *d*′ was calculated by defining the hit rate as the proportion of true items correctly endorsed, and the false positive rate as the proportion of lures incorrectly endorsed, and then using the formula:

$$d^{\prime }\;=\;\text{qnorm}\left(\text{hit}\;\text{rate}\right)\;–\;\text{qnorm}\left(\text{false}\;\text{positive}\;\text{rate}\right)$$

where qnorm is the z‐transform [Pallier, 2002]. In addition, we calculated a *d*′ measure reflecting the overall discrimination of listed items versus non‐listed items, by changing the false positive rate to include all unpresented items. Finally, we simply calculated the proportion of critical lures falsely endorsed. No outliers were found in this task.

## Results

### Visual Extrapolation Tasks

**Figure 2:**
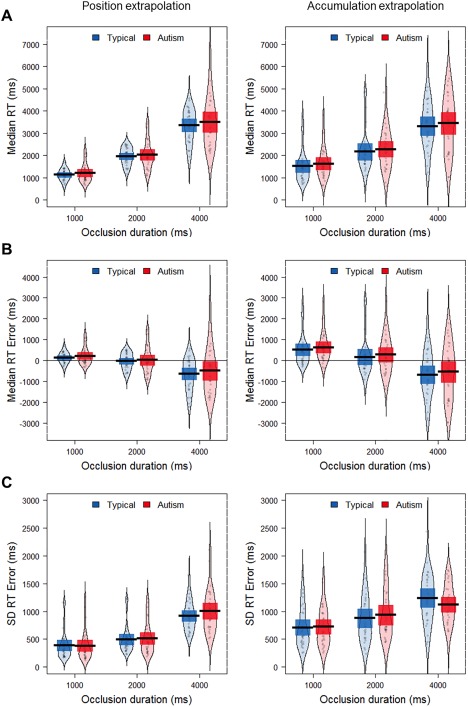
Visual extrapolation task performance for typically developing and autistic children. Individual participants' median RT (**A**), median RT error (**B**) and standard deviation of RT error (**C**) for each occlusion duration, with group means (black lines), 95% confidence intervals (dark colored bands) and smoothed density.

Figure 2 shows children's median RTs, median RT errors and standard deviation of RT errors in the visual extrapolation tasks. In line with Makin and Bertamini [2014], RTs increased as a function of occlusion duration (Fig. 2A), and there was a tendency for children to overestimate the time at the shortest occlusion duration (positive RT errors) and underestimate at the longest occlusion duration (negative RT errors) (Fig. 2B).

To test our first hypothesis that autistic children would make less precise predictions than typically developing children, we analyzed the standard deviation of RT errors (Fig. 2C). We conducted a mixed ANOVA for each task, with occlusion duration as a within‐participants factor and group as a between‐participants factor. The results confirmed that our difficulty manipulation had the expected effect, with longer occlusion durations leading to more variable performance in both the position, *F*(2,116) = 123.81, *P* < 0.001, *η*_p_^2^ = .68, and accumulation, *F*(2,116) = 42.44, *P* < .001, *η*_p_^2^ = 0.42, tasks. Repeated contrasts showed that performance was significantly more variable in the 2000 ms occlusion duration than the 1000 ms occlusion duration, and more variable in the 4000 ms occlusion duration than the 2000 ms duration, in both tasks, *P* ≤ 0.002. However, there were no significant main effects of group in either the position, *F*(1,58) = .26, *P* = 0.62, *η*_p_^2^ < .01, or accumulation, *F*(1,58) = .03, *P* = 0.87, *η*_p_^2^ < .01, tasks, and no interaction between group and occlusion duration (position: *F*(2,116) = 0.72, *P* = 0.49, *η*_p_^2^ = .01; accumulation: *F*(2,116) = 1.59, *P* = 0.21, *η*_p_^2^ = .03).

To determine whether there was sufficient evidence for the null hypothesis (of no group differences) relative to the alternative hypothesis of higher standard deviations in the autism group, we conducted one‐sided Bayesian independent samples *t*‐tests using JASP software [JASP Team, 2017] with a default Cauchy prior width of 0.707 [Wagenmakers et al., 2017]. In all cases, there was relatively more evidence in support of the null hypothesis than the alternative hypothesis (inverse Bayes Factors [BF_01_] > 1), although the strength of this evidence varied across conditions (see Table 2). BF_01_ above 3 represents substantial support for the null hypothesis, whereas below 3 represents weak evidence [Jeffreys, 1961]. Thus, there was substantial support for the null hypothesis in the position task at the two shortest occlusion durations and in the accumulation task for the shortest and longest durations. The data provided only weak support for the null hypothesis in the longest duration for the position task and the middle duration for the accumulation task, suggesting that more data is required before drawing a firm conclusion in these conditions. Robustness checks are provided in Supplementary Material to assess the influence of the prior. Although not mentioned in the preregistration, we also conducted Bayesian repeated measures ANOVAs (with JASP's default prior) and found substantial evidence for the null hypothesis in both the position (BF_01_ = 4.59) and accumulation (BF_01_ = 3.99) tasks across occlusion durations.

**Table 2:** Results of One‐Sided Bayesian Independent Samples *t*‐tests for the Visual Extrapolation Tasks

	Inverse Bayes Factors (BF_01_)	
Occlusion duration	Position	Accumulation
1000 ms	4.03	3.46
2000 ms	3.22	2.54
4000 ms	1.60	7.22

*Note*. BF_01_ refers to the relative evidence for the null hypothesis over the alternative hypothesis that autistic children have more variable performance than typically developing children.

Further, exploratory analyses found neither significant main effects of gender (*P* ≥ 0.45) nor interaction effects involving gender (*P* ≥ 0.06) when added as an additional factor in the ANOVA. Verbal IQ was negatively correlated with overall standard deviation in the position task (across all occlusion duration), with higher IQ values linked with less variable RTs, *r*(58) = −.31, *P* = 0.02. We repeated the ANOVAs with verbal IQ as a covariate but found that this did not change the pattern of results. Additional exploratory analyses showed that overall standard deviation of RTs was not significantly correlated with age, SCQ or ADOS severity scores (*P* ≥ 0.08). Finally, we found no significant effects of group nor interactions between group and occlusion duration (*P* ≥ 0.53) in median RT error, but a significant effect of occlusion duration on all measures (*P* < 0.001).

### False Memory Task

**Figure 3:**
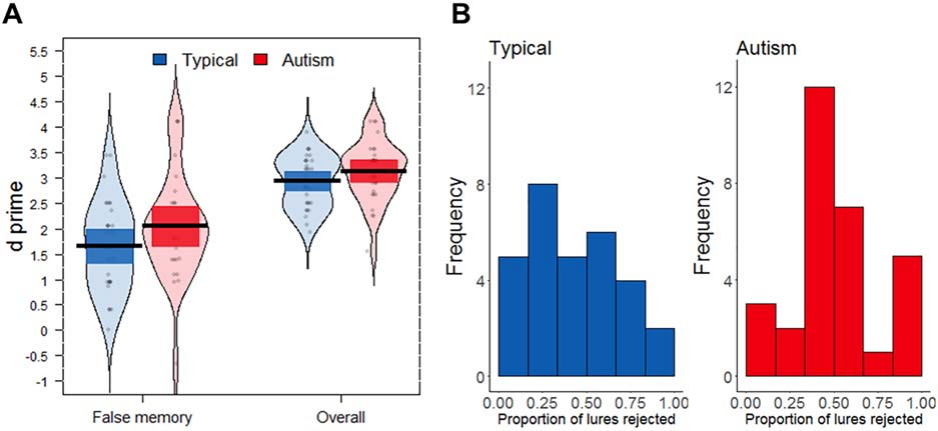
False memory performance for typically developing and autistic children. (**A**) D prime for discriminating previously presented items from critical lures (“false memory”) and for discriminating previously presented items from all non‐presented items (“overall”). Shown are the group mean (black line), 95% confidence intervals (dark colored bands), individual data points and smoothed density. (**B**) Histograms for the proportion of critical lures correctly rejected for each group.

Our second hypothesis was that autistic children would be less prone to false memories than typically developing children. A one‐sided independent samples t‐test on false memory *d*′ (i.e., discrimination of true listed items vs. lure items; Fig. 3A) found a non‐significant difference between the two groups (typically developing: *M* = 1.66, SD = .89; autistic: *M* = 2.05, SD = 1.02), *t*(58) = 1.55, one‐sided *P* = 0.06, *d* = .40. A one‐sided Bayesian *t*‐test with a Cauchy prior width of .65 gave a BF_01_ of 0.73, meaning that the alternative hypothesis of group differences was 1.37 (1/0.73) times more likely than the null hypothesis, representing weak evidence [Jeffreys, 1961] in support of the alternative hypothesis that autistic children are better at discriminating true items from critical lure items (see robustness checks in Supplementary Material).

For a more general test of sensitivity, we also investigated *d*′ across all listed vs. non‐listed items. Again, the group difference was not significant (typically developing: *M* = 2.93, SD = .52; autistic: *M* = 3.13, SD = .59), *t*(58) = 1.37, one‐sided *P* = 0.09, *d* = .35. The BF_01_ was 0.93, indicating little evidence for either the null or alternative hypothesis. Finally, Figure 3B shows the proportion of lures correctly rejected. Most children, in both groups, demonstrated false memories for at least one critical lure item. Here too, the groups did not differ significantly (typically developing: *M* = 0.51, SD = 0.26; autistic: *M* = 0.58, SD = .25), *t*(58) = 1.16, one‐sided *P* = 0.13, *d* = 0.30, corresponding to a BF_01_ of 1.20 suggesting weak evidence in favor of the null hypothesis.

Exploratory analyses confirmed that verbal IQ was not correlated significantly with performance (*P* ≥ 0.42). We also tested whether gender differences were contributing to our results, finding no significant effect of gender or interactions between group and gender for any false memory measure (*P* ≥ 0.24). We next conducted exploratory analyses to investigate the effect of age, as it has been suggested that autistic and typically developing children may diverge in false memory susceptibility as they get older [Miller et al., 2014]. None of the false memory measures were significantly correlated with age, in either group, *P* ≥ 0.16. Additionally, false memory measures were not significantly correlated with the SCQ or ADOS severity scores, *P* ≥ 0.28.

### Relationship between Visual Extrapolation and False Memory Tasks

Our third and final hypothesis was that accurate discrimination would be associated with poor performance on the extrapolation tasks. We tested this with partial correlations between overall standard deviation of RT error (across all occlusion durations) and false memory measures, controlling for age and performance IQ. Opposite to prediction, false memory *d*′ was significantly negatively correlated with standard deviation in the accumulation task, *r*(56) = −.29, *P* = 0.03, and correlated in the same direction, but not significantly, in the position task, *r*(56) = −.21, *P* = 0.11. The overall *d*′ measure was similarly correlated with standard deviation in both the accumulation, *r*(56) = −.38, *P* = 0.003, and position, *r*(56) = −.33, *P* = 0.01, tasks, but the proportion of lures rejected was not (accumulation: *r*(56) = −.18, *P* = 0.18; position: *r*(56) = −.09, *P* = 0.51).

## Discussion

We presented visual extrapolation and verbal false memory tasks to autistic and typically developing children and found no evidence for predictive impairments in the autistic children. Autistic children predicted the end‐point of occluded objects as well as typically developing children. The groups did not show clear differences in the false memory task, either, although there was weak evidence for autistic children being better at discriminating between presented and non‐presented items than typically developing children. Insofar as there were relationships between performance in the two tasks, they were opposite to our hypothesis, with children who were more sensitive in the false memory task showing less variable performance in the extrapolation tasks.

We used visual extrapolation tasks to test Sinha et al. [2014]'s assertion that autistic individuals have difficulties anticipating moving objects. Our data showed no such deficit, either for predicting the motion of a single moving target or the accumulation of multiple elements. We also predicted that autistic children would show particular difficulties when required to extrapolate over longer occlusion durations [Sinha et al., 2014]. In all occlusion conditions apart from two, we found substantial evidence for the null hypothesis. Weak evidence was obtained for the null hypothesis in the longest occlusion condition of the position task, but substantial evidence for the null was obtained in the longest occlusion condition for the accumulation task, opposing our hypothesis of more pronounced group differences at longer durations.

Could children succeed by using predictive eye movements in this task? These were shown to be unimpaired in a previous study of perception of moving targets in autistic individuals [Ego et al., 2016]. Information from eye movements could be used in the position extrapolation task, but this is far less plausible for the accumulation task. Instead, both extrapolation tasks are believed to reflect the updating of mental representations using a common‐rate controller [Makin & Bertamini, 2014], as well as visual processing, temporal processing, sustained attention, and the execution of a motor response. Our tasks involved making predictions where there was a lawful relationship between the visible motion and the end‐point of occluded motion. Perhaps it is unsurprising that autistic children were able to make predictions in these tasks, given that basic motion processing is generally found to be unimpaired or even enhanced in autism [Foss‐Feig, Tadin, Schauder, & Cascio, 2013; Manning, Tibber, Charman, Dakin, & Pellicano, 2015; see Simmons et al., 2009 for review]. We conclude that the basic predictive mechanisms involved in these relatively ‘low‐level’ perceptual tasks are not impaired in autistic children. These results pose challenges for theories suggesting pervasive impairments in prediction [Sinha et al., 2014].

Arguably, prediction abilities should not be considered as a single entity and instead theories should consider the underlying taxonomy of prediction abilities. Difficulties with prediction may become apparent when anticipating objects with more complex motion trajectories, or when making predictions between events that are more weakly associated, such as those involving social information [Balsters et al., 2017; von der Lühe, Manera, Becchio, Vogeley, & Schilbach, 2016; Sevgi, Diaconescu, Tittgemeyer, & Schilbach, 2016]. Additionally autistic individuals may have difficulties when required to decide what information is relevant—an aspect of real‐world prediction that may not be captured by highly controlled experimental tasks. This suggestion appears to fit well with the hierarchical quality of the predictive coding framework, which could potentially explain why some types of prediction are more difficult for autistic individuals than others. This notion also echoes recent work into atypical adaptive mechanisms in autism, which suggest that adaptation to “low‐level” stimuli, like perceptual causality and color, may be intact in autism [Maule, Stanworth, Pellicano, & Franklin, 2016; Karaminis et al., 2015], whereas reduced adaptation is found for more “high‐level” stimuli, such as faces and numerosity [Pellicano et al., 2007; Turi et al., 2015]. We note that previous studies have often focused on hypotheses arising from predictive coding and Bayesian accounts [e.g., Lawson et al., 2017; Sevgi et al., 2016] rather than testing prediction abilities directly. It remains a challenge for future research to link these approaches in order to understand how altered predictive mechanisms at the level of the brain translate to predictions made in the world. Additionally, it is important for theories to be specified appropriately so that they can be robustly falsified.

Our study also adds to the literature assessing false memory performance in autism. The false memory illusion is a robust effect in the general population [Zwaan et al., 2017], but the evidence for reduced susceptibility to false memories in people with autism is less compelling, with Hillier et al. [2007] and Bowler et al. [2000] both failing to replicate the effect in verbal false memory tasks. Our study offers some insight into these apparently conflicting results. Our power analysis showed that the minimum sample size to detect an effect size of *d* = 0.65 for a power of 80% and an alpha of .05 was 30 participants per group. Yet, most studies of false memory in autism have used far fewer than this. Moreover, the true effect size may be smaller than this [Anderson, Kelley, & Maxwell, 2017], as it was in the current study (*d* = 0.40), necessitating even larger samples. While the groups were not significantly different in our study, our Bayesian analyses showed that the data were insensitive to discriminate between the null and alternative hypotheses, with slightly more evidence in favor of the alternative hypothesis of group differences. Therefore, we do not rule out superior false memory performance in autistic individuals, but suggest that the effect may be relatively small. Moreover, while we used a recognition task, group differences may have become apparent in a less supported free recall task [Bowler, Matthews, & Gardiner, 1997]. In the same vein, the position and accumulation extrapolation tasks also provided structure (the track and grid) to help participants formulate their responses, and it is possible that autistic children may have shown predictive difficulties had these supports not been available.

Our rationale for presenting these seemingly different tasks together was that theories proposing disordered prediction in autism are closely linked to theories proposing reduced use of context or ‘gist’. We predicted that *reduced* performance in the visual extrapolation task would be related to *increased* sensitivity in the false memory task, but we found the opposite. This relationship could reflect domain‐general factors (e.g., motivation or attentiveness) or the involvement of a memory component. Future research with cross‐task comparisons will therefore be necessary to elucidate links between prediction abilities and contextual processing in autism.

In summary, we provide evidence of preserved prediction abilities in autistic children for dynamic objects, suggesting that prediction abilities are not generally impaired in autistic children. Future research will be needed to characterize the nature of predictive impairments in autism. Our results are compatible with superior false memory performance in autistic children, but larger samples will be needed to provide conclusive evidence. Our results therefore help to refine theories of both altered prediction and contextual processing in autism.

## Supporting information

Supporting Information
