# Supplementary material for: Visual Motion Prediction and Verbal False Memory Performance in Autistic Children
Source: Autism Res. 2017 Dec 21;11(3):509–18. doi: 10.1002/aur.1915 (PMC5901411; doi:10.1002/aur.1915)
Supplement: Supplementary file 1 — Supporting Information [file AUR-11-509-s001.docx]

**Supplementary Material**

**Word lists for the false memory task**

|  | **List 1** | **List 2** | **List 3** | **List 4** | **List 5** | **List 6** |
| --- | --- | --- | --- | --- | --- | --- |
| **Word list** | Apple  Orange  Banana  Food  Grape  Strawberry  Pear  Juice | Bed  Pillow  Dream  Covers  Night  Tired  Nap  Sheets | Candy  Sugar  Chocolate  Ice cream  Taste  Nice  Sour  Cookies | Toes  Shoes  Walking  Socks  Jumping  Ankle  Leg  Running | Wheel  Petrol*  Window  Radio  Seat  Engine  Steering  Drive | Nurse  Shot  Medicine  Checkup  Surgery  Patient  Sick  Help |
| **Critical lure** | Fruit | Sleep | Sweet | Foot | Car | Doctor |
| **True** | Apple  Strawberry | Bed  Night | Candy  Taste | Toes  Socks | Wheel  Radio | Nurse  Sick |
| **Distant false** | Bowl  Basket | Peace  Yawn | Tart  Heart | Mouth  Smell | Road  Bus | Cure  Office |
| **Unrelated false** | Coal  Sister | Small  Bench | Kitten  Royal | Knitting  Old | Art  Ground | Shutter  Toast |

* The word ‘gas’ used by Metzger et al. (2008) was substituted for ‘petrol’ for British English-speaking children.

Note. The order of word presentation was counterbalanced across children.

**Robustness checks for Bayesian independent samples t-tests: Visual extrapolation tasks**

| **Occlusion Duration** | **Position** | **Accumulation** |
| --- | --- | --- |
| 1000 ms | 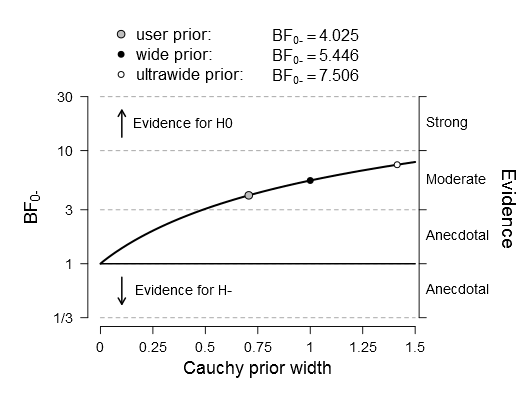 | 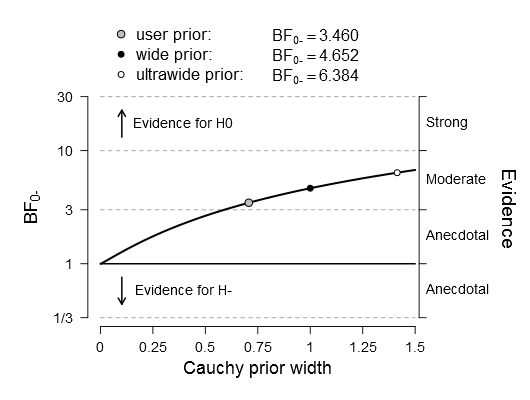 |
| 2000 ms | 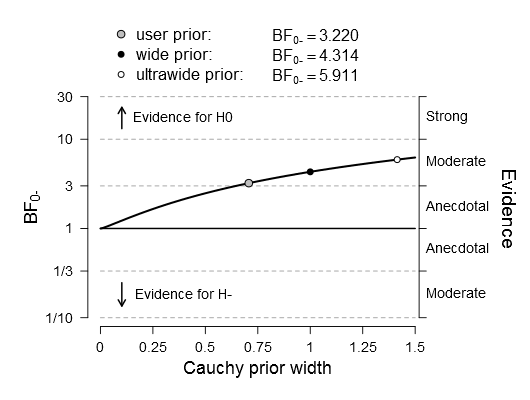 | 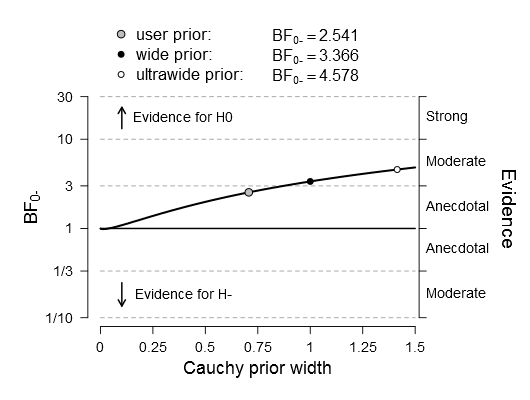 |
| 4000 ms | 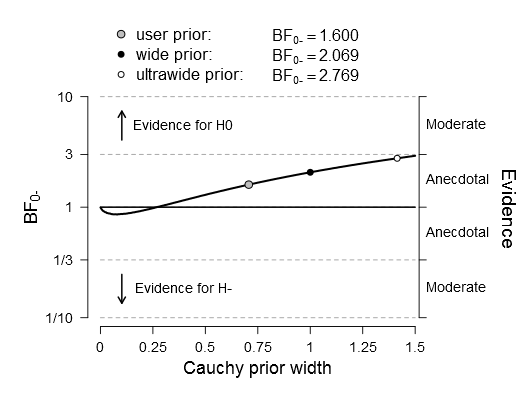 | 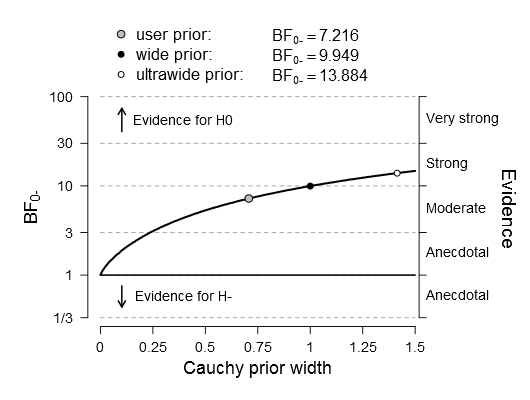 |

**Robustness checks for Bayesian independent samples t-tests: False memory task**

| False memory d’ | 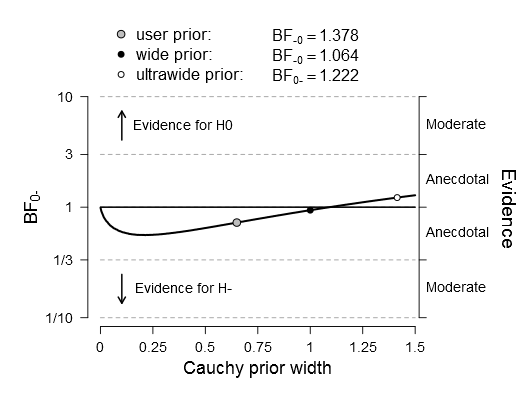 |
| --- | --- |
| Overall d’ | 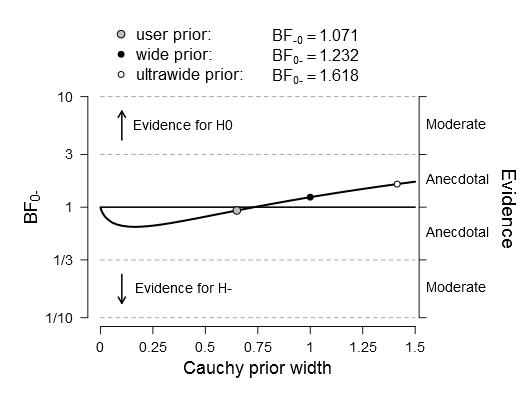 |
| Critical lure rejection | 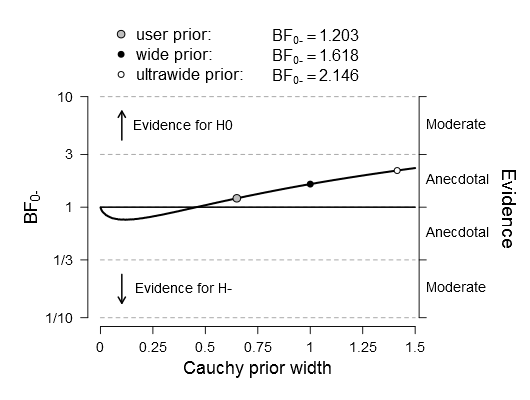 |
